# Integrating Climate Change Adaptation into Public Health Practice: Using Adaptive Management to Increase Adaptive Capacity and Build Resilience

**DOI:** 10.1289/ehp.1103515

**Published:** 2011-10-13

**Authors:** Jeremy J. Hess, Julia Z. McDowell, George Luber

**Affiliations:** 1 Climate and Health Program, Division of Environmental Hazards and Health Effects, National Center for Environmental Health, Centers for Disease Control and Prevention, Atlanta, Georgia, USA; 2 Department of Environmental Health, Rollins School of Public Health, and; 3 Department of Emergency Medicine, Emory University School of Medicine, Emory University, Atlanta, Georgia, USA

**Keywords:** adaptive management, climate change, climate change adaptation, public health, public health administration

## Abstract

Background: Climate change is expected to have a range of health impacts, some of which are already apparent. Public health adaptation is imperative, but there has been little discussion of how to increase adaptive capacity and resilience in public health systems.

Objectives: We explored possible explanations for the lack of work on adaptive capacity, outline climate–health challenges that may lie outside public health’s coping range, and consider changes in practice that could increase public health’s adaptive capacity.

Methods: We conducted a substantive, interdisciplinary literature review focused on climate change adaptation in public health, social learning, and management of socioeconomic systems exhibiting dynamic complexity.

Discussion: There are two competing views of how public health should engage climate change adaptation. Perspectives differ on whether climate change will primarily amplify existing hazards, requiring enhancement of existing public health functions, or present categorically distinct threats requiring innovative management strategies. In some contexts, distinctly climate-sensitive health threats may overwhelm public health’s adaptive capacity. Addressing these threats will require increased emphasis on institutional learning, innovative management strategies, and new and improved tools. Adaptive management, an iterative framework that embraces uncertainty, uses modeling, and integrates learning, may be a useful approach. We illustrate its application to extreme heat in an urban setting.

Conclusions: Increasing public health capacity will be necessary for certain climate–health threats. Focusing efforts to increase adaptive capacity in specific areas, promoting institutional learning, embracing adaptive management, and developing tools to facilitate these processes are important priorities and can improve the resilience of local public health systems to climate change.

## Public Health Adaptation to Climate Change

Many potential climate change health impacts have been established, and several are already evident (McMichael et al. 2004). Climate change is expected to increase the burden of climate-sensitive diseases such as heat-related illness, vector-borne disease, diarrheal disease, injuries from extreme events, and respiratory diseases (Campbell-Lendrum and Woodruff 2006). Although the developing world is most at risk (Patz et al. 2007), industrialized countries are also ill prepared (Ebi et al. 2009; Maibach et al. 2008; O’Neill et al. 2010). Indeed, the event with the most dramatic health impact attributed to climate change thus far, the European heat wave of 2003, occurred in the ostensibly well-prepared industrialized world, illustrating the disastrous effects of extreme weather made more likely by climate change (Stott et al. 2004), by a relatively unprepared public health sector [World Health Organization (WHO) 2009], and by high levels of both population exposure (Poumadere et al. 2005) and susceptibility (Rey et al. 2009).

Public health institutions at all operational scales will need to consciously modify their approaches to both science and practice in anticipation of climate change health impacts, and much has been written on various aspects of these issues. Several articles have outlined climate change as a public health concern, advancing the public health community’s awareness (Frumkin et al. 2008; Haines et al. 2006; Patz et al. 2005). Others have explored climate change epidemiology and risk assessment (Campbell-Lendrum and Woodruff 2006; Kovats et al. 2005; McMichael 2001) by examining fundamental scientific questions and by providing epistemological insights. Still others have clarified methodologies and practical strategies for conducting vulnerability and impact assessments (Ebi et al. 2006), assessed relevant environmental health frameworks (Füssel 2008), and articulated guidelines for climate impact and adaptation assessments and advanced research agendas (Portier et al. 2010).

Despite this progress, however, with notable exceptions (Ebi and Burton 2008; Ebi and Semenza 2008; Ebi et al. 2005, 2006; Huang et al. 2011; Jackson and Shields 2008), there has been little discussion of how public health organizations should implement and manage the process of planned adaptation. Huang et al. (2011) note that this includes both enhancing adaptive capacity, that is, the resources for adaptation and the ability to use them effectively and efficiently, and implementing adaptive actions. They also offer several suggestions for overcoming likely barriers. Apart from this significant work, however, there has been relatively little discussion of how to increase public health’s adaptive capacity or how this process could increase public health’s resilience.

Literature from other sectors provides some general guidance for building adaptive capacity. Besides resource availability, many other factors are important, including social and human capital, attention to institutional decision making and information management, and processes for spreading risk (Yohe and Tol 2002). Also key is the promotion of social learning, which means building collective knowledge through social interactions (Berkhout et al. 2006; Pahl-Wostl 2009) and integrating learning into management (McDaniels and Gregory 2004; Social Learning Group 2001). Other literature highlights the connection between high levels of adaptive capacity and resilience in socioeconomic systems, emphasizing that such systems have the capacity to retain their essential structure and function after significant disruption, to reorganize, and to learn (Folke 2006). Despite their relevance to public health adaptation, these insights have not been fully synthesized for the public health context specifically.

This article uses a substantive, interdisciplinary literature review to identify strategies for expanding public health’s adaptive capacity through an emphasis on learning and changes in management frameworks. Our review explores possible explanations for the relative dearth of work on adaptive capacity in public health and potential implications for policy and practice; highlights several climate-sensitive health threats that may overwhelm public health’s adaptive capacity; reviews the role of learning in building adaptive capacity; and considers how adaptive management—a strategy that integrates learning and management—might increase adaptive capacity, thereby fostering the development of more resilient public health systems.

## Perspectives on Public Health’s Adaptive Capacity

Apart from highlighting the need for additional resources in many settings, adaptive capacity has not been a major focus of the climate–health literature. There are two possible explanations, each based on a particular take on climate change as a public health stressor. Reviewing these explanations lends insight into differing perspectives on how best to build adaptive capacity and facilitate public health adaptation.

The first explanation is that climate change is not likely to require substantial changes to public health practice other than increased investment and program expansion, that is, increasing resources and implementing adaptive measures are the primary means of increasing adaptive capacity. This perspective holds that climate change will primarily amplify known public health stressors, which affect vulnerable populations most dramatically. Because human health is already heavily managed via extensive infrastructure (Füssel 2008), it follows that established practices will likely be sufficient if given the appropriate mandate, adequate funding, and support. By extension, effective adaptation will be characterized primarily by investments that reinforce essential public health services (Frumkin et al. 2008). This perspective affirms public health’s readiness contingent on sufficient support and puts the emphasis on bolstering rather than reconfiguring public health practice.

The second explanation is that innovations in public health practice are likely necessary to enhance adaptive capacity, but a broad literature base that supports this contention is not yet available. This dearth of literature may be because innovative strategies have not yet materialized in many locations, perhaps because adaptation tends to occur in response to the stimulus of extreme events (Berrang-Ford et al. 2011), or because such strategies have not yet made their way into the literature. The contention that innovative strategies will be required is based on a concern that climate change, which could jeopardize critical infrastructure and destabilize various systems that maintain public health, may represent a categorically distinct public health stressor. Thus, novel frameworks, strategies, and tools are required to help manage systemic risk. Rather than affirming the conventional approach that adaptation will primarily entail program expansion, the innovation perspective, recognizing its limitations, highlights its limits, particularly the potential for systemic instability to undermine public health gains. In focusing on potential failures, this innovation-oriented view emphasizes novel management strategies in addition to standard public health programming to enhance adaptive capacity.

Although these two narratives are not mutually exclusive, there is tension between them, for several reasons. First, the two perspectives lead to different funding priorities. Given current budget constraints, funding new initiatives is likely to come at the expense of other programming, and investments without clear near-term payoffs are hard to sell. Even in settings with a well-developed infrastructure, climate change adaptation competes, often unsuccessfully, with other urgent public health concerns (Ebi et al. 2009; Maibach et al. 2008). Second, particularly in regions with less public health infrastructure, many believe that adaptation should be secondary to more immediate concerns, such as basic public health services and essential medicines. Third, most public health institutions and health care systems have chosen to rely on existing infrastructure and all-hazards preparedness rather than investing in innovations when increased risks have yet to materialize (Hess et al. 2009). Fourth, a management-oriented, systems-based, long-view approach to public health is logistically difficult to pursue because it requires secure long-term funding, interdisciplinary and intersectoral collaboration, and integrated information management, which existing funding and administrative structures inadvertently discourage (Füssel 2008; Haines et al. 2009).

## Identifying Public Health Impacts That Exhibit Distinct Climate Sensitivity

Determining the relative merit of the two perspectives is a primary challenge for public health practitioners interested in increasing adaptive capacity and developing resilient public health systems. Because climatic stressors, population vulnerabilities, and public health capacities are variably distributed, this determination will be context specific (Hess et al. 2008). Although multiple guidelines can help clarify needs in particular contexts, however, none satisfactorily addresses the full range of policy questions (Füssel 2008), and there are no criteria to help determine how to portion investments between bolstering current activities and developing innovative programming.

Identifying areas where vulnerability is particularly high—threats that exhibit distinct climate sensitivity—can help clarify where efforts to increase adaptive capacity should be focused. Criteria for identifying these threats include

High population vulnerability to hydrometeorological hazards (i.e., high levels of exposure and susceptibility and low adaptive capacity), such that increases in the frequency and severity of such hazards will significantly increase overall risk (Ebi et al. 2006; Keim 2008; Schneider et al. 2007). One example is systems in which recurrent flooding, combined with other exposures that erode household coping capacity (O’Brien and Leichenko 2000; Webster and Jian 2011), undermines long-term adaptive capacity and increases cumulative risk (Tapsell et al. 2002).
The potential for extreme events associated with climate change to present hazards outside the coping range (Yohe and Tol 2002) of a given public health system. The probability of the European heat wave of 2003, for instance, was significantly increased by anthropogenic emissions (Stone et al. 2009; Stott et al. 2004) and by imposed stresses outside the coping capacity of the public health system (Lagadec 2004; Poumadere et al. 2005).
The likelihood that increasingly severe and frequent hazards associated with climate change could undermine or compromise systems and infrastructure and have significant population health impacts (Gerber 2007; McDaniels et al. 2008). For example, more frequent heat waves increase reliance on mechanical air conditioning, increasing electricity demand and thus the probability of cascading grid failure.
The likelihood that climate change will fundamentally alter basic ecosystem services important to public health (Myers and Patz 2009; Schroter et al. 2005). Examples are abundant, including ecosystem shifts driving increased bioaccumulation of toxins such as mercury and polychlorinated biphenyls (Carrie et al. 2010) and the potential for groundwater salinity as a result of saltwater intrusion from sea level rise (Khan et al. 2008).
The likelihood that climate change will result in abrupt ecosystem shifts (Walther 2010) favoring the introduction or reemergence of diseases for which effective surveillance and management practices are not yet in place. An example of this is the 2004 outbreak of *Vibrio parahaemolyticus* associated with Alaskan oysters harvested during an unusually warm period, which abruptly shifted the northernmost range of the endemic area for this disease by 1,000 km (McLaughlin et al. 2005).

Applying these criteria to major public health concerns, for example, tobacco use, teen pregnancy, and health-care–associated infections, it is clear that many do not currently exhibit distinct climate sensitivity and are not likely to in the near future. Although climate change may affect the distribution of some health outcomes that are not distinctly climate sensitive (e.g., road traffic injuries may worsen with changes in precipitation), bolstering existing programming may well be sufficient to address these shifting threats. Similarly, in lower resource settings, emphasizing basic service provision will likely be most strategic. In moderate to high resource settings, however, other strategies to enhance adaptive capacity may be more strategic.

## Arguments for Focusing on Distinctly Climate-Sensitive Threats

For several reasons, identifying public health challenges exhibiting distinct climate sensitivity is important for building adaptive capacity in settings where basic needs are already addressed.

First, it will focus effort on the subset of problems requiring substantial innovation and collaboration, and this focus can help address known barriers to adaptation. Other sectors have identified a need for focused innovative adaptation efforts (Smithers and Blay-Palmer 2001), and health is likely to be similar. Indeed, public health has previously identified challenges in need of focused innovation, such as the articulation of the Grand Challenges in Global Health and corresponding funding of innovative strategies to address these challenges (Cohen 2005; Varmus et al. 2003). As other sectors have shown, some catalytic innovations—which use novel technologies or strategies to bring goods or services to whole new populations—can result in both improved population outcomes and lower costs (Christensen et al. 2006), an appealing prospect in a time of worsening budgetary constraints.

Second, a focused approach could minimize friction between the climate–health community and other areas of public health. Although this friction has not proven a significant impediment to date, there are several instances—such as the contentious debate around climate change and malaria (Chaves and Koenraadt 2010; Reiter 2001)—in which the emphasis on climate change has been seen as an inappropriate distraction from established, evidence-based efforts at disease prevention and control.

Third, such a focus may be strategic from a policy perspective, because it allows climate–health advocates to highlight the need for general investment in public health infrastructure, particularly in resource-poor settings, where “adaptation to climate change is essentially a matter of basic public health protection” (Campbell-Lendrum et al. 2007), as well as specific climate–health programming for issues of greatest concern. This may prove attractive to policy makers who craft health adaptation portfolios in the developing world, where a strong case can be made for general investment in public health to reduce climate-related and other risks.

## Management Challenges for Distinctly Climate-Sensitive Public Health Concerns

Developing effective adaptations to distinctly climate-sensitive health threats presents a host of management challenges, including uncertainty in climate projections and future socioeconomic conditions; financial challenges and other maldistribution of existing adaptive capacity; limits in technological advancement and dissemination; institutional arrangements that limit the scope of collaborative efforts and accumulation of evidence about effective adaptation; limits on social capital at the community level; and uninformed or inaccurate perceptions of individual risk (Huang et al. 2011). Two other issues, scale and complexity, are also significant.

The scale issues that complicate adaptation are both temporal and spatial. Temporal concerns include the need to focus on short-term planning for discrete events, such as a severe heat wave, and longer-term needs for strategies to reduce hazardous exposures and increase resilience (McMichael and Dear 2010; Sherwood and Huber 2010). Spatial concerns arise from mismatches between hazard distributions, political and administrative boundaries, and resource availability. The issue of spatial scale and climate has been explored more thoroughly in the ecological (Clarke et al. 2007; Seo et al. 2009), agricultural (Baron et al. 2005), and modeling (Diffenbaugh et al. 2005) literature than in public health, although examinations of heat hazards at various scales (Harlan et al. 2006; Stone et al. 2010) and synchrony of cholera outbreaks (Constantin de Magny et al. 2007) suggest how this research may unfold.

Complexity is perhaps the most pervasive concern. Huang et al. (2011) note one aspect of this issue in their discussion of limits to individual cognition and risk perception. The issue of complexity extends well beyond individual cognition, however, to a host of systems concerns related to managed socioecosystems, from cities to fisheries, whose complex dynamics, including delays, positive and negative feedbacks, stock-and-flow relationships (Sterman 2000, 2008), and thresholds (Codeco et al. 2008), complicate management. Climate change has introduced additional uncertainty into these dynamics and highlighted the need for new strategies to understand and manage such systems, emphasizing the need for an approach that fully captures impacts and facilitates informed management (Grabs et al. 2007; Howden et al. 2007). This echoes a general trend toward systems-based investigation in environmental health (Gohlke and Portier 2007) and public health in general (Diez Roux 2011), risk management (Bea et al. 2009), ecology (Montoya and Raffaelli 2010), and economics (Polasky and Segerson 2009). Recent operations research in public health has also come to similar conclusions about other aspects of the public health system (Van Wave et al. 2010), insights that need to be applied to climate change adaptation.

## The Role of Learning

These management challenges highlight the need for strategies that embrace uncertainty and emphasize learning (Sterman 2006). Scholarly work on learning theories, such as experiential learning (Kolb 1984) and transformative learning (Mezirow 1995), emphasize concrete learning cycles, learning by doing, and the ways learning feeds into reinterpretation of value structures. The learning loop framework (Argyris and Schön 1978) integrates these insights and divides learning into three categories based on the extent to which the learning promotes transformative change in management strategies.

Single-loop learning focuses on improving the efficiency of action by reconciling differences between what is expected and what is observed (Pelling et al. 2007), for example, whether a dike is high enough to contain anticipated flooding. Double-loop learning considers whether management strategies are appropriate (Flood and Romm 1996), for example, whether dikes are the most appropriate strategy in the context of changing precipitation distributions. Triple-loop learning examines underlying principles and value systems (Pelling et al. 2007) and power relationships (Flood and Romm 1996) to explore the range of possible management options, for example, new approaches to governance, participatory risk management, and planning aimed at robust actions instead of strategies that are optimal for particular constituents or conditions (Pahl-Wostl 2009).

Each type of learning is relevant for public health adaptation. There are a host of strategies for facilitating institutional learning and for incorporating learning into management (Armitage et al. 2008). In particular, the adaptive management framework is a potentially useful approach for increasing adaptive capacity by increasing learning at all levels and reorienting management approaches to distinctly climate-sensitive health threats.

## Adaptive Management and Its Potential

Adaptive management was developed as an iterative method for managing natural resource systems where linear approaches had failed (Holling 1978) as a result of the systems’ wide range of responses to management choices, the managers’ difficulty understanding the systems’ dynamics (Linkov et al. 2006), and the dynamic interplay between managers, stakeholders, interventions, and system responses (Henriksen and Barlebo 2008). To manage these systems, ecosystem managers needed an iterative process that acknowledged complexity and uncertainty, emphasized ongoing learning, and allowed for continuous stakeholder input. Adaptive management was created in response to these needs (Whicker et al. 2008).

**Figure 1:**
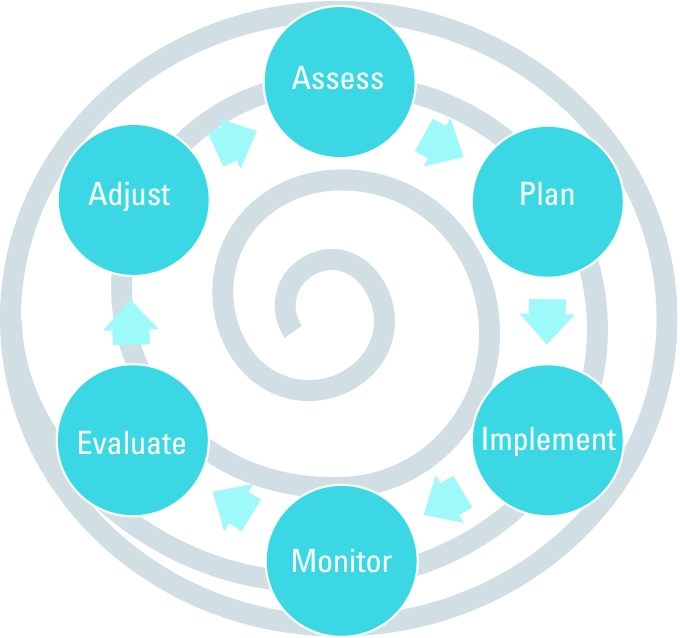
The adaptive management cycle.The steps in the process are shown in the circles, the arrows indicate the direction of the process flow, and the central spiral emphasizes the goal of arriving at a robust consensus based on a shared set of objectives developed through the iterative process. Adapted from Whicker et al. (2008).

The National Research Council’s (2004) guide to adaptive management emphasizes six primary elements: *a*) management objectives that are regularly revisited and revised, *b*) a model of the system(s) being managed, *c*) a range of management choices, *d*) monitoring and evaluation of outcomes, *e*) mechanisms for incorporating learning into future decisions, and *f*) a collaborative structure for stakeholder participation and learning. These steps are diagrammed in Figure 1. The process allows for an approach tailored to the unique specifics of each system and situation and integrates management and learning instead of consigning them to different domains (Murray and Marmorek 2003).

Adaptive management has yet to secure a significant place in the public health toolbox, although several agencies have used it to engage a wide range of environmental health concerns, sometimes coupled with structured decision analysis processes (Linkov et al. 2006). Adaptive management has been difficult to implement in certain instances, although a systematic review suggests that difficulties primarily stem from application of the framework in inappropriate contexts (Gregory et al. 2006). This review suggests that adaptive management is most appropriate in circumstances in which modeling and decision-making scales are matched and external factors are considered, there is explicit consideration of uncertainties, stakeholders agree on metrics of cost and risk, and stakeholders are sufficiently engaged and provide adequate institutional support.

In regard to climate change, as Ebi (2011) has noted, adaptive management closely parallels frameworks for general climate change adaptation (Lim et al. 2005) and public health adaptation (Ebi and Semenza 2008). It has been used to explore issues related to ecosystem management (Prato 2010), watersheds (Pulwarty and Melis 2001), emissions trading (Satterstrom et al. 2007), and air quality monitoring (Stubbs and Lemon 2001). In its “active” form, which facilitates analysis of multiple decision possibilities, adaptive management appears to have significant potential for public health adaptation efforts, particularly at the local to regional scale.

## Adaptive Management of Distinctly Climate-Sensitive Health Threats

Many of the essentials of adaptive management—modeling complex, dynamic problems; interacting with a wide range of stakeholders; and an evidence-based, iterative approach to decision making—are familiar to public health. The process is perhaps most akin to evidence-based medicine and its cousin, evidence-based public health (Brownson et al. 2009; Eriksson 2000). As with these approaches, embracing the entire paradigm confers several advantages over a disjointed approach.

The potential of adaptive management and the tools required are perhaps best conveyed through an example. Of the various hazards associated with climate change, extreme heat events (EHEs) are the best studied and among the most urgent. Although considerable uncertainty regarding heat morbidity remains, we have a solid understanding of heat–mortality functions (Basu 2009; Ishigami et al. 2008; Kovats et al. 2008; Pirard et al. 2005) and a rapidly evolving understanding of the factors that put populations at risk, from physiological susceptibility (Ellis 1972, 1976; Kilbourne et al. 1982) to exposure (Basu 2009; Harlan et al. 2006; Ishigami et al. 2008; Reid et al. 2009) to aspects of the built (Clarke 1972; Sheridan and Dolney 2003; Silva et al. 2010; Stone et al. 2010) and social environments (Klinenberg 2002; Rey et al. 2009), as well as a sense of several successful interventions at multiple levels (Hajat et al. 2010a, 2010b; O’Neill et al. 2009, 2010; Semenza 2006; Sheridan 2006; Silva et al. 2010; WHO 2009).

Models are fundamental to adaptive management and can be relatively straightforward conceptual models that distill the system into key components or more complex computer-based models (Ebi 2011). Integrated assessment models (IAMs) are examples of the latter and are often used to facilitate decision making and to assess impacts of potential interventions. These models draw from multiple disciplines to capture system behavior (Chan et al. 1999). Such frameworks have been developed only for certain climate-sensitive health outcomes, and there are currently no IAMs for heat specifically, although some models of urban heat impacts are being developed (Dawson et al. 2009). Team-based modeling efforts to organize and focus group thinking are also relevant (Vennix 1996). Examples of these types of models, and other tools, are presented in Table 1.

**Table 1:** Steps in the adaptive management cycle, central actions in each step, and tools useful for completing the central actions.

Adaptive management step	Action	Existing tools	Example	Additional tools needed
Assess		Estimate likelihood and severity of exposure currently and in the future		Impact assessment		FEMA Hazus software (FEMA 2011)		Assessment tools to incorporate downscaled climate projections
						MIASMA Health Impact Assessment (Tizio BV/Netherlands Environmental Assessment Agency 2011)		
		Gauge susceptibility of population to hazard, including social components of vulnerability		Vulnerability assessment		UNFCCC/WHO Health Vulnerability Guidelines (Kovats et al. 2003)		Better quantitative vulnerability assessment methods that can be projected
Plan		Prioritize high-risk populations and areas for response		Vulnerability mapping		California Vulnerability Map (California Department of Public Health 2009)		Easily accessible mapping software with wider geographic coverage
				Hazard mapping		Puerto Rico Disaster Tool (University of Delaware, Disaster Research Center 2011)		
		Formulate politically and economically feasible response plan		Adaptation options compendia		Center for Climate and Energy Solutions 2011		Models to predict effectiveness of given adaptation decisions
				Decision support tools		Adaptation Decision Matrix (Stratus Consulting 2007)		
		Evaluate cross-sectoral needs under emergency circumstances		Integrated assessment model		Tyndall Center Urban Integrated Assessment Facility (Dawson et al. 2009)		Cross-sectoral models and other tools to avoid cascading impacts
Implement		Communicate preparedness and response and plans to stakeholders		Early warning systems		Philadelphia heat early warning system (Ebi et al. 2004)		Improved tools for communicating risk to the public
Monitor		Capture data relevant to expected impacts and interventions		Syndromic surveillance		CDC Syndromic Surveillance (Henning 2004)		Better systems to capture and process data in real time
				Remote sensing		NASA data for heat early warning system (Johnson 2011)		
Evaluate		Compare pre- and postassessments or two similar events		General M&E guidelines		UNFCCC guidance for monitoring and evaluation of adaptation (UNFCCC 2010)		Quantitative methods to manage uncertainty and changing conditions
Adjust		Change management approach based on evaluation, changing future conditions, stakeholder input		Problem-based learning		Adaptive management activities in the natural resources sector (Bryan et al. 2009)		Tools to facilitate ongoing stakeholder engagement and multicriteria decision analysis
Abbreviations: FEMA, Federal Emergency Management Association; M&E, Monitoring and Evaluation; MIASMA, Modeling Framework for the Health Impact Assessment of Man-Induced Atmospheric Changes; UNFCCC, United Nations Framework Convention on Climate Change.								

In the case of heat, the urban environment is a particularly relevant system, for several reasons: most of the world’s population now lives in cities (United Nations Population Programme 2004); cities have high concentrations of people vulnerable to heat-related injury (Campbell-Lendrum and Corvalán 2007; Hess et al. 2008); urban environments amplify heat exposure at several levels (Campbell-Lendrum and Corvalán 2007; Patz et al. 2005; Stone et al. 2010); EHE response plans are typically administered at the metropolitan level (Bernard and McGeehin 2004); and municipal health authorities are often underprepared for EHEs (Bernard and McGeehin 2004; O’Neill et al. 2010).

Despite the lack of an IAM for urban heat, we can outline an adaptive management process focused on EHEs and consider how this process might evolve iteratively as uncertainties regarding the climate system, health communications, exposure determinants, population susceptibility, and the response to various potential interventions are clarified.

**Figure 2:**
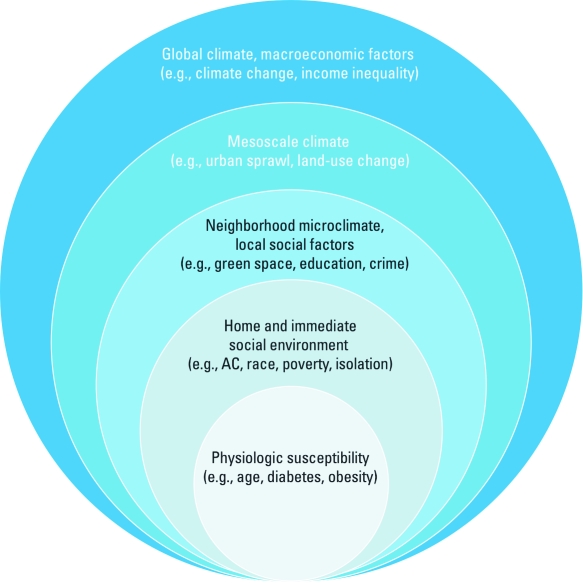
Components of heat-related morbidity and mortality risk operative at various spatial scales. AC, air conditioning.

*Assessment.* Assessment is the first step of the adaptive management process (Figure 1). This is one type of vulnerability assessment, for which multiple theoretical frameworks and methodologies are available. In the case of heat, several components of risk, from hazard frequency and severity to population exposure and susceptibility, must be assessed. EHE risk results from the interaction of various factors at multiple scales, as depicted in Figure 2. Using the natural hazards risk formula to incorporate hazard probability, hazard exposure, and population susceptibility (Malilay et al. 1997), taking care to incorporate social factors affecting vulnerability (Sullivan and Meigh 2005), can help organize these components. A wide range of stakeholders should be engaged, from neighborhoods to emergency medical responders to city planners to electrical and water utilities, in order to assess dynamics affecting both exposure and response. Substantial literature provides insight into effective strategies for stakeholder engagement (Lim et al. 2005).

*Planning.* Planning prepares for real-world implementation and often uses IAMs. Response activities incorporated into the model should parallel exposures, that is, strategies to change land use and urban form at the mesoscale (Clarke 1972; Golden 2004; Shimoda 2003); building materials, vegetation, and other factors affecting sensible heat at the neighborhood and street levels (Jenerette et al. 2007; Silva et al. 2010); home visitation and other social capital strategies at the neighborhood level (Luber and McGeehin 2008; Wolf et al. 2010); and strategies for changing the home and other environments and relocation of susceptible people (O’Neill et al. 2005). Planning should also incorporate a range of possible futures and be tailored to stakeholder inputs. Improved forecasts that are downscaled to a finer geographic scale can help to limit uncertainty.

Certain tools allow practitioners to organize information on the hazard and population at risk in order to prioritize responses. Vulnerability mapping, for example, allows for visual rendering of relative population vulnerability in relation to hazards and response infrastructure (Li et al. 2010; Morrow 1999). The maps should be used to identify a range of possible interventions to incorporate into the IAM. Decision support tools, including software tools, documents, and work processes, are designed to help practitioners and policy makers evaluate decisions available to them and the potential impacts of those decisions across complex systems, but few tools for selecting adaptation options are available (Pyke et al. 2007).

Stakeholders should also heavily influence the selection of adaptation options. Adaptation requires a new level of cross-sectoral planning, and other sectors are increasingly acknowledging the need to incorporate health (Kashyap 2004) and vice versa (Cole et al. 2007). In the case of extreme heat, electricity generation for air conditioning is a primary concern, and water and forestry are also important. Dynamic models to simulate such interconnected relationships have not been well developed in public health but are increasingly important. Adaptive management must consider scenarios in which other sectors that typically facilitate public health are not fully functional, and alternatives must be modeled and explored. Importantly, research has shown that the primary threat to such systems is the inability of managers to reorganize and recover from significant stressors (Bodin and Norberg 2005; Bunce et al. 2009), highlighting the role of intersectoral collaboration and communication in the planning process.

*Implementation.* Implementation occurs at various time, geographic, and administrative scales. For instance, implementation of strategies focused on hard infrastructure (e.g., changes in the built environment) will occur at longer time scales than those focused on changes in vegetation, outreach programs, and implementation of early warning systems. From an administrative perspective, implementation will take place via established networks, although adaptive management should result in more interdisciplinary, transsectoral implementation efforts. Implementation will require integration of several dynamic information streams tracking exposures (Webster and Jian 2011), population response to early warnings (Basher 2006; Ebi and Schmier 2005; Kashyap 2004), and assets available for response. A wide variety of decisions must be made at different administrative levels (Luber and McGeehin 2008), such as how predictions will be made, what variables will be tracked, how warnings will be conveyed, thresholds for triggering warning messages (Hajat et al. 2010b; Metzger et al. 2010), and strategies for acting on preparedness plans (Balbus et al. 2008) and communicating warnings (Ebi 2007).

*Monitoring.* Monitoring provides data fundamental to learning in adaptive management (Holling 1978). Monitoring should be planned early in the process (Ebi 2011) and capture relevant data. Monitoring for EHE management would ideally capture shifts in exposures and modifying factors at various levels, changes in demographics, urban form, and outcome, such as heat morbidity and mortality rates. Syndromic surveillance of symptoms of heat-related illness can be analyzed in real time, for instance, to detect significant increases in these symptoms even before diagnoses are confirmed and reported to public health agencies, facilitating earlier response and ongoing changes in tactics as an outbreak progresses (Josseran et al. 2009). Other exposure indicators are also important, sometimes using remote sensing (Johnson et al. 2009). Other longer-term indicators should be tracked at larger geographic and administrative scales, if possible. In the United States, this might include health indicators that are or soon will be tracked at state and national levels (English et al. 2009). Particular attention should be paid to vulnerable populations (Balbus and Malina 2009). Monitoring should also capture system interactions and capacity. For instance, both short- and medium-term electrical power generation capacity are important determinants of EHE adaptation; although utilities monitor capacity, there is little coordination to increase public health preparedness.

*Evaluation.* Evaluation in adaptive management is explicitly focused both on the efficacy of the intervention (management objectives) and on increasing understanding of the system being managed (learning objectives) (Satterstrom et al. 2007). This introduces the need for statistical support of pre- to postassessments in an iterative process, often involving Bayesian frameworks (Henriksen and Barlebo 2008). Such pre- to postassessment is fundamentally probabilistic and requires both managers and stakeholders be educated on this approach, although it is often intuitive even for stakeholders without significant specific training (Webster and Jian 2011).

Carrying through the extreme heat example, several issues can complicate evaluation efforts. Often multiple interventions are mounted concurrently, as was the case after the European heat wave of 2003, making it difficult to parse their relative contributions. Moreover, because of constantly shifting baseline conditions, it is difficult to generate baseline estimates of disease burden. However, comparing one extreme event with another can give some indication of efficacy, as with the 2003 and 2006 heat waves in Europe, where the later heat wave resulted in far lower mortality after significant prevention measures were taken (Fouillet et al. 2008).

*Adjustment.* Adjustment is crucial to adaptive management. The adjustment phase is when future decisions regarding management and research are made, linking to the next cycle (Figure 1). During adjustment, stakeholders are again actively engaged, results of the initial management decisions are conveyed, and stakeholders and system managers convey input regarding the next cycle. Adjustment is thus a process of information synthesis and communication as well as enhanced decision making and the point at which significant learning occurs (Bormann et al. 2007). Adjustment also has important implications for the social integration of stakeholders, which has been shown to improve resilience to climate change in other sectors (Tompkins and Adger 2004).

Adjustment is also where the cycle is at greatest risk. Reviews of adaptive management efforts have shown that inattention to key social learning elements—particularly rapid knowledge acquisition, effective information management, and explicit attention to creating shared understandings among diverse stakeholders—are key culprits (McLain and Lee 1996). This is a concern in any discipline, but public health, with its emphasis on the social determinants of health and integration within community based organizations, has a set of tools for facilitating such processes (Baker et al. 2005; Rowitz 2004). Coupled with appropriate tools for managing information flow within and between organizations and a strong stakeholder commitment to the process, these tools are crucial for the adjustment phase.

## Tools to Facilitate Adaptive Management

Many tools are available to facilitate adaptive management (Table 1), falling into three categories: assessment tools for identifying and locating hazards and vulnerable populations; tools to model, project, or evaluate specific climate-related health threats using scenarios; and decision support tools to evaluate adaptation options. In addition to these three categories, it will be crucial to refine tools for evaluating public health adaptive management efforts, for which several methods are available (McFadden et al. 2011), and for performing cost–benefit analyses of adaptive management efforts. Currently there is no comprehensive, centralized tool repository, although such a resource could maximize diffusion of innovations.

## Conclusion

To date much of the climate–health literature has focused on establishing and projecting climate change health impacts. This work has shown that certain distinctly climate-sensitive health threats are very likely to pose challenges outside public health’s coping range. The question of how to increase public health capacity has received less attention. Our findings suggest that management of these threats is likely to require innovative strategies acknowledging that the systems protecting public health have limited resources and are dynamic, incompletely understood, and subject to multiple stakeholders. Institutional learning at multiple levels is key to increasing adaptive capacity, and adaptive management is a potentially useful framework. Its components are familiar, but the coordinated process and the use of modeling in iterative decision making are relatively new. Several helpful tools are available but must be revised for new contexts, and significant gaps remain (Table 1). Developing a centralized tool repository should be a high priority and, along with increased focus on learning, modeling, and adaptive management, will help increase the resilience of local public health systems.
